# The shifting income-obesity relationship: Conditioning effects from economic development and globalization

**DOI:** 10.1016/j.ssmph.2021.100849

**Published:** 2021-06-18

**Authors:** Min Zhou

**Affiliations:** Department of Sociology, University of Victoria, Cornett Building A359, 3800 Finnerty Road, Victoria, BC, V8W 3P5, Canada

**Keywords:** Obesity, Health, Income, Globalization, Multilevel model

## Abstract

The literature has long been debating whether it is high-income or low-income individuals who face higher risks of obesity. In this study I contend that this mixed record about the income-obesity relationship is the result of a failure to account fully for macro-level social contexts. The income-obesity relationship is not uniform in all societies but is conditioned by macro-level social contexts including the society's economic development and involvement in globalization. The 2011 Module on Health and Health Care of the International Social Survey Programme (ISSP) provides an ideal opportunity for testing the complex income-obesity relationship in a cross-country setting. Employing multilevel models with cross-level interactions, this study finds that the shift in the effect of income from obesity-promoting to obesity-depressing is facilitated by both economic development and globalization. Under the combined forces of economic development and globalization, obesity increasingly becomes a burden of the poor in a society and the social distribution of obesity increasingly mirrors existing social inequality. Nevertheless, the economic development and globalization thresholds for shifting into a significant obesity-depressing effect of income are high.

## Highlights

• Economic development weakens the obesity-promoting effect of income.
• Globalization weakens the obesity-promoting effect of income.
• Income promotes or is not associated with obesity in most countries.
• Income depresses obesity only in highly developed and globalized countries.

## Introduction

Obesity is defined as a disease by the World Health Organization (WHO), because it significantly increases the risk of many chronic diseases such as cardiovascular disease, hypertension, some diabetes, chronic kidney disease, and many cancers (WHO 2000, 2005). Obesity not only leads to high health care costs for the family and the whole society (Finkelstein et al., 2003), but also negatively impacts individuals' life in many ways. Obese individuals report lower general wellbeing (Stewart et al., 2009), face more challenges in the labor market (Han et al., 2009), and are more prone to discrimination and cultural stigma (Brewis, 2011; Farrell, 2011). Realizing these individual and social consequences of obesity, social scientists have been keen on finding out social determinants of obesity.

While obesity is partially influenced by genetic features, it also has a social dimension. Obesity is not evenly distributed in society, but its distribution can be shaped by individuals' socioeconomic status such as income. While scholars generally agree that there can be a relationship between income and obesity, they have been debating whether it is high-income or low-income individuals who face higher risks of obesity and empirical results in the literature have been mixed (Marteleto et al., 2017; McLaren, 2007; Monteiro et al., 2004; Pampel et al., 2012; Powell et al., 2012; Sobal & Stunkard, 1989; Villar & Quintana-Domeque, 2009; Zhang & Wang, 2007). In this study I contend that this mixed record about the income-obesity relationship is the result of a failure to account fully for macro-level social contexts. The income-obesity relationship is not uniform in all societies, but is conditioned by macro-level social contexts such as the society's economic development and involvement in globalization.

First, the income-obesity relationship can be contingent on a country's economic development. Studies of individual countries often find that income and obesity are positively associated in less developed countries but negatively associated in developed countries (Dinsa et al., 2012; Marteleto et al., 2017; Monteiro et al., 2004; Zhou, 2019). In less developed countries those with higher income are more likely to be obese, whereas it is those with lower income who face higher risks of obesity in developed countries. This observation leads to “the reversal hypothesis” about the income-obesity relationship—as the economy of a country develops, the relationship between income and obesity risks shifts from positive to negative (Pampel et al., 2012). Economic development potentially induces changes such as changing diet, labor market structure, and culture about body shape that may reshape the income-obesity relationship in a society. What is missing in this reversal hypothesis is that we do not know exactly at what levels of economic development the income-obesity relationship shifts directions in a country. This study attempts to identify the GDP per capita threshold for shifting the effect of income from obesity-promoting to obesity-depressing. Moreover, this reversal hypothesis also fails to disentangle the influences of a country's economic development and involvement in globalization. We do not know to what extent the shift in the income-obesity relationship can be attributed to economic development after the influence of globalization is taken into account.

Second, the level of a society's integration into globalization may also condition the income-obesity relationship in this society. Although globalization is considered to be a driving force of the rapid rise in obesity over the globe (Costa-Font & Mas, 2016; De Vogli 2013; Goryakin et al., 2015; Hawkes, 2006; Miljkovic et al., 2015; Popkin, 2006), no empirical studies have investigated the impact of globalization on the income-obesity relationship. Countries are integrated into globalization to different degrees, which in turn affects the income-obesity relationship in particular countries. New moral assumptions and popular discourses about obesity have emerged and become prominent in the world culture, which have given rise to negative assessments of obesity or even social stigma (Austin, 1999; Brewis, 2011; Brewis et al., 2011; Coveney, 2000; Farrell, 2011). Globalization diffuses cultural perceptions of the ideal slim body shape through cultural presentations embodied in popular culture, mass media, and marketing (Bordo, 2003; Calogero et al., 2007; Farrell, 2011). Social groups are not affected by this world culture in the same way, however. It is normally wealthy individuals who enjoy more global cultural products (such as foreign movies, magazines, commercials, and overseas travels) and are thus more exposed to global cultural influences (Vassilakopoulou & Hustad, 2021). Hence, compared with those in less globalized countries, affluent individuals in more globalized countries may develop a stronger cultural aversion to obesity and income can have a greater depressing effect on obesity. This study is the first empirical study that investigates how integration into globalization conditions the income-obesity relationship in a country.

Taken together, through examining the effect of income on obesity that is contingent on the society's economic development and involvement in globalization, this study aims to reveal under what social conditions income has a promoting or depressing effect on obesity. This differential income-obesity relationship is theoretically interesting because it helps unpack the link between social inequality and obesity. While high-income individuals have more resources to cope with negative consequences of obesity, obesity aggravates difficulties facing poor individuals. If low-income individuals have higher risks of obesity in a society, obesity would exacerbate existing social inequality. Through investigating the social contexts that condition the income-obesity relationship, this study sheds light on the type of society in which obesity is more prone to engender greater social inequality.

### The contingent nature of the income-obesity relationship

Income has been demonstrated to influence the risk of obesity because it affects individuals' energy intake and expenditure and also shapes their access to health-related resources, knowledge, and skills (Marteleto et al., 2017; Martin et al., 2012; Villar & Quintana-Domeque, 2009; Wang, 2001; Zhang & Wang, 2007). This income-obesity relationship can be contingent on both the country's economic development and its integration into globalization, however.

### Economic development

Most previous studies on socioeconomic determinants of obesity have examined the association between income and obesity in developed countries, the United States and European countries in particular. They generally suggest a negative relationship between income and obesity in the population or subpopulation. A few studies have begun investigating the income-obesity relationship in developing countries (Dinsa et al., 2012; Marteleto et al., 2017; Monteiro et al., 2004; (Zhou, 2019)). They find income and obesity can be not or positively associated in developing countries (McLaren, 2007; Monteiro et al., 2004; Pampel et al., 2012; Sobal & Stunkard, 1989; (Zhou, 2019)). These studies point to a potential shift in the direction of the income-obesity relationship induced by economic development, or the reversal hypothesis (Pampel et al., 2012).

Some theoretical support for this reversal hypothesis can be found in the health literature. Nutrition transition theory (Drewnowski & Popkin, 1997; Popkin, 2001; Popkin et al., 2002) contends that economic development gives rise to a shift from a diet mainly featuring grains and vegetables to a diet composed of more animal-sourced products and energy-dense industrially produced foods. Given the nutrition transition brought about by economic development, socioeconomic disparities in obesity in a society can be heavily dependent on the stage of economic development. In developing countries where food insecurity is still a challenge, low income “limits the resources available for excess food consumption” and increases the necessity of physically demanding labor, whereas high income makes possible “both access to excess food and avoidance of labor-demanding work” (Pampel et al., 2012, p. 1074). Heavy weight may even serve as a status marker (Brewis, 2011). A big size can be perceived as a symbol of higher social status.

To the contrary, in developed countries where the economy has shifted to service sectors and technology industries, most have sufficient food security and are able to avoid labor-demanding work (Brownson et al., 2005). Inexpensive high-calorie foods become more widely available, and occupations require much less physical labor. These changes reshape the relationship between income and obesity (Bleich et al., 2008; Cutler et al., 2003). Overall, low-income individuals face a greater risk of obesity because they tend to consume more high-calorie foods that are cheaper. In contrast, healthy foods such as those high in fiber and low in calories are often more expensive, and their production cannot be easily industrialized. High-income individuals can better afford them (Cawley, 2004).

Taken together, as a country's economy (GDP per capita as a proxy) becomes more developed, the obesity-promoting effect of income is weakened and even turns into an obesity-depressing effect. Going beyond this hypothesis, I further aim to reveal at what levels of GDP per capita the effect of income shifts directions.

Hypothesis 1 A country's economic development (GDP per capita) weakens the obesity-promoting effect of income.

### Globalization

A considerable literature has realized the connection between globalization and obesity and generally found an obesity-promoting effect of globalization (Costa-Font & Mas, 2016; De Vogli et al., 2013; Goryakin et al., 2015; Hawkes, 2006; Miljkovic et al., 2015; Popkin, 2006). Globalization has the potential to both increase energy consumption and lower energy expenditure. It leads to more abundant supply and consumption of cheaper processed foods higher in calories, and creates more opportunities for enjoying lifestyles with decreased energy expenditure (such as more use of cars and more indoor activities). Going beyond the direct obesity-promoting effect of globalization found in the literature, I further propose that the extent of a country's integration into globalization conditions the income-obesity relationship in the country.

In recent years, a world culture that values a thin body shape has emerged and become increasingly dominant (Bordo, 2003; Brewis et al., 2011; Calogero et al., 2007). This world culture consists of both a cultural perception about the beautiful body and a health discourse that stresses obesity as a primary source of many diseases. Globalization is currently dominated by cultural influences from the developed Western world (Beckfield, 2003) and disseminates the Western standard of beauty through ubiquitous messages for beauty in advertising, mass media, and the entertainment industry (Swami et al., 2010). This global culture only values a certain physical stereotype of beauty, and one key component of the beauty perception is body weight. This beauty perception is closely related to the health discourse that highlights the importance of health associated with thinness (Butler-Wall, 2016). In this health discourse, weight is believed to be controllable and obese people are often portrayed as lacking self-discipline (Blaine & McElroy, 2002; Crandall & Martínez, 1996). A thin body shape is considered to be both beautiful and healthy in the globalized world culture. The beauty perception and the health discourse impact individuals' body attitudes and behaviors. Because of increasing global access to Western cultural representations, there is a global trend toward a homogenization of the thin ideal body image (Austin, 1999; Coveney, 2000).

Globalization facilitates the penetration of these cultural norms and perceptions into a country. Usually it is wealthy individuals in a society who are more exposed to these global influences. They have more access to and consume more foreign cultural products such as movies and magazines and also travel overseas more frequently, so they are more exposed to the world culture (Vassilakopoulou & Hustad, 2021). The globalized beauty perception and health discourse view obesity as unhealthy and undesirable, and associate obesity with lower classes and social stigma that wealthy individuals try to avoid. With more wealth at their disposal, wealthy individuals are well equipped with resources to achieve a slender or fit body. They also tend to employ the globalized beauty perception and health discourse to legitimate and reinforce class distinctions (Guthman, 2009). Accordingly, more integration into globalization is expected to depress obesity among wealthy individuals more than among poor ones, thereby facilitating the shift of the positive income-obesity relationship toward a negative one in a country.

Hypothesis 2 A country's level of globalization weakens the obesity-promoting effect of income.

## Data and method

The data are from the 2011 Module on Health and Health Care of the International Social Survey Programme (ISSP) (available at http://www.issp.org). The 2011 wave of the ISSP collected rich data on various health-related issues in multiple countries, thereby providing a rare opportunity for testing the complex income-obesity relationship across countries. Altogether there are 49,987 individuals from 30 countries in the data. The data cover 30 countries including Australia, Belgium, Bulgaria, Chile, China, Croatia, the Czech Republic, Denmark, Finland, France, Germany, Israel, Italy, Japan, South Korea, Lithuania, the Netherlands, Norway, the Philippines, Poland, Portugal, Russia, the Slovak Republic, Slovenia, Spain, Sweden, Switzerland, Turkey, the United Kingdom, and the United States. Although the 2011 wave of the ISSP also included South Africa and Taiwan, they are excluded in the analysis due to them missing data on key variables. To the ISSP data I also append country-level measures from other data sources. The data are structured hierarchically, with 49,987 individuals at Level 1 nested within 30 countries at Level 2. Individuals' characteristics are located at Level 1, whereas the country's economic development and integration into globalization are located at Level 2.

### Dependent variable

Following the convention recommended by the health literature and the WHO, I use an individual's body mass index (BMI) to measure the dependent variable obesity. BMI is defined as an individual's weight in kilograms divided by the square of the same individual's height in meters. The WHO recommends using BMI ≥30 as the cut-off point for identifying obesity in adults (WHO 2000). According to the WTO, a BMI of 30 or higher signals a medical condition in which excess body fat has accumulated to the extent that it has an adverse effect on health. This cut-off point is found to be effective for identifying obesity-related risk factors including hypertension, diabetes, and cardiovascular diseases. The ISSP data contain information on individuals' weight and height. I calculate each individual's BMI and then define obesity as BMI ≥30. The dependent variable is binary, with 1 indicating obesity and 0 not.

**Fig. 1:**
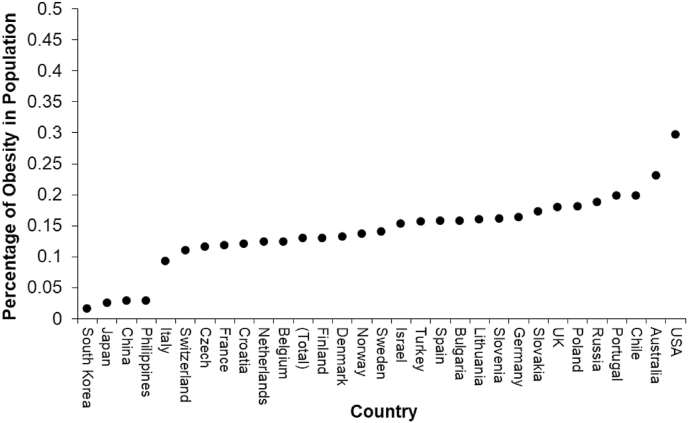
Levels of obesity (percentages of obesity in the population) across countries.

Fig. 1 depicts the distribution of obesity across countries. The level of obesity varies greatly across countries. The percentage of obese people in the national population ranges from 1.64 percent (South Korea) to 29.67 percent (the USA). The global average is 12.92 percent.

### Country-level independent variables

The first country-level explanatory variable is national economic development. GDP per capita in US dollars is a conventional proxy. To make cross-country comparisons, I use GDP per capita in terms of purchasing power parity constant US dollars (PPP$). Data are drawn from the World Bank Database (available at http://databank.worldbank.org/data/home).

The second country-level explanatory variable is the country’ level of globalization. Data are from the KOF Index of Globalization (available at http://globalization.kof.ethz.ch). The KOF globalization index is a composite indicator that summarizes a country's integration into globalization along economic, political, and cultural dimensions (Dreher, 2006; Dreher et al., 2008; Gygli et al., 2018). It represents arguably the most systematic measure of globalization across countries and has been widely used in empirical studies (see Potrafke, 2015) including those on the impact of globalization on obesity (Costa-Font & Mas, 2016; De Vogli et al., 2013; Goryakin et al., 2015).

### Individual-level independent variables

The individual-level explanatory variables include income, gender, education, age (and age squared), place of residence, working status, and marital status. These characteristics are seen as relevant to an individual's risk of obesity, and are thus commonly used as explanatory variables (Ljungvall & Gerdtham, 2010; Pampel et al., 2012; Villar & Quintana-Domeque, 2009; (Zhou, 2019)).

Income is measured by the respondent's monthly per capita household income (i.e., household income divided by the number of people in the household) converted into constant US dollars. Gender is a binary variable, with male coded as 1 and female as 0. Education is measured by the years of education received. Age is measured in years. The effect of age may not be perfectly linear, so I also include a quadratic term of age in the analysis. Location of residence is a dummy variable, in which rural residence (including “a country village” and “a farm or home in the country”) is coded as 1 and urban residence (including “a big city,” “the suburb or outskirt of a big city,” and “a town or a small city”) as 0. Working status has three categories including currently working, having formerly worked but currently not working, and having never worked. I create three dummy variables and use currently working as the reference group in the analysis. Marital status contains four categories including single (never married), married, divorced or separated, and widowed. Four dummy variables are generated and the single (never married) category is used as the reference group. I also tried including the variable of self-placement on a 10-point scale (measured by this survey question “In our society, there are groups which tend to be towards the top and groups which tend to be towards the bottom. Below is a scale that runs from the top to the bottom. Where would you put yourself on this scale?”). Including this variable does not substantively change the findings below.

Table 1 displays basic descriptive statistics about the variables used in the analysis.

**Table 1:** Descriptive statistics for variables used in the analysis.

Variable	Mean	Std. Dev.	Minimum	Maximum
Dependent variable				
Obesity	.129	.335	0	1
Independent variables at the country level				
GDP per capita	30,283.920	14,997.850	5690	62,077
Globalization	78.112	10.594	56.97	91.97
Independent variables at the individual level				
Income	1207.932	2644.738	0	171,917
Gender (male)	.458	.498	0	1
Education	11.916	4.503	0	35
Age	48.496	16.958	15	100
Residence (rural)	.348	.476	0	1
Working status				
Currently working	.567	.496	0	1
Formerly worked	.348	.476	0	1
Never worked	.085	.279	0	1
Marital status				
Single	.218	.413	0	1
Married	.611	.487	0	1
Divorced (incl. separated)	.089	.285	0	1
Widowed	.082	.274	0	1

Notes: Female is the reference group for the gender variable (male = 1; female = 0); urban is the reference group for the residence variable (rural = 1; urban = 0).

### Model

Multilevel logistic regression is used in the analysis. The multilevel model can accommodate the multilevel nature of the research question and the multilevel structure of the data, while the binary nature of the dependent variable requires logistic regression. I define *P*_*ij*_ as the probability of respondent *i* being obese in country *j* and let *P*_*ij*_ be modeled using a logit link function. Then the two-level random-intercept model can be specified as follows:

Log[P_ij_/(1 − P_ij_)] = β_1j_Income_ij_ + β_2j_Male_ij_ + β_3j_Education_ij_ + β_4j_Age_ij_ + β_5j_Age^2^_ij_ + β_6j_Rural_ij_ + β_7j_Work_ij_ + β_8j_Marital_ij_ + γ_01_GDP_j_ + γ_02_Globalization_j_ + γ_00_ + u_0j_ + ε_ij_

where β and γ are the coefficients of the individual-level and country-level variables, respectively. γ_00_ is the intercept, which is allowed to vary across countries. u_0j_ estimates this random effect. ε_ij_ is the error term.

To test the effect of income on obesity contingent on GDP per capita (Hypothesis 1) and globalization (Hypothesis 2), to the basic model above I add two cross-level interaction terms, Income × GDP per capita and Income × Globalization, respectively. I further allow the coefficient of income (β_*1j*_) to vary across countries and estimate a series of two-level random-slope models. Note that the random part of the random-slope models now becomes the segment *u*_*0j*_*+ u*_*1j*_ *Income*_*ij*_ + *ε*_*ij*_. While the random-intercept models assume the same coefficient of income across countries, the random-slope models allow the coefficient of income to vary across countries.

I employ the *melogit* command in the Stata software (release 16) (StataCorp 2019) to estimate these multilevel logistic models. For each model I also calculate the Akaike information criterion (AIC) and Bayesian information criterion (BIC) values as model fit statistics (Akaike, 1974; Raftery, 1995). The AIC and BIC are widely used indicators for the goodness of model fit. When several models are estimated with the same data, the one with the smaller value of the information criterion is considered to be better. I utilize the post-estimation command *estat ic* in Stata to calculate the AIC and BIC.

## Results

### Individual-level and country-level influences on obesity

Table 2 presents the multilevel logistic regression models that examine the individual- and country-level influences on the risk of obesity. Each model consists of two parts—a fixed-effects part that shows estimated coefficients and a random-effects part that presents the variance component of the intercept and the slope across countries (thereby capturing other uncontrolled variances across countries). Model fit statistics are reported at the bottom.

**Table 2:** Multilevel logistic models of obesity.

	Model 1	Model 2	Model 3	Model 4
Fixed effects				
Intercept	−1.966*** (.132)	−4.882*** (.299)	−8.893*** (1.195)	−9.459*** (1.284)
Individual-level variables				
Income (in thousands)		-.0001 (.0002)	-.0001 (.0001)	-.0002 (.0002)
Gender (male)		.032 (.031)	.032 (.031)	.032 (.031)
Education		-.052*** (.004)	-.052*** (.004)	-.052*** (.004)
Age		.142*** (.006)	.142*** (.006)	.142*** (.006)
Age^2^		-.0013*** (.0001)	-.0013*** (.0001)	-.0013*** (.0001)
Residence (rural)		.082* (.033)	.080* (.033)	.080* (.033)
Working status				
Currently working		Reference	Reference	Reference
Formerly worked		.374*** (.037)	.374*** (.037)	.376*** (.037)
Never worked		.308*** (.066)	.310*** (.066)	.312*** (.066)
Marital status				
Single		Reference	Reference	Reference
Married		-.072 (.046)	-.071 (.046)	-.071 (.046)
Divorced		-.288*** (.063)	-.286*** (.063)	-.286*** (.063)
Widowed		.177* (.068)	.181** (.068)	.181** (.068)
Country-level variables				
GDP per capita (in thousands)			-.009 (.013)	-.012 (.013)
Globalization			.054** (.018)	.065*** (.018)
Random effects (variance across countries)				
Intercept	Included	Included	Included	Included
Slope (income)				Included
LR test	1756.6***	1889.7***	1361.4***	1361.8***
Model fit statistics				
Wald χ^2^	–	1062.7***	1075.0***	1100.8***
AIC	32719.6	31082.7	31075.1	31074.7
BIC	32737.0	31195.7	31189.5	31185.1

Notes: (1) Numbers in parentheses are standard errors; (2) from 2-tailed tests, *P < .05; **P < .01; ***P < .001; (3) under “Random Effects” the LR test reports the likelihood ratio statistic for testing the null hypothesis that the random effects (between-country variance) are zero. A significant test result indicates that it is necessary to include random effects in the modeling.

I begin with a baseline model (Model 1) with only an intercept and its random effects. The intercept is −1.966, which translates into a predicted probability of 0.129. On average, across all countries 12.9 percent of the population is obese. It is worth noting that the random effects in Model 1, as well as in all other models in Table 2, are statistically significant, so the intercept does vary across countries. I also calculate the intraclass correlation (ICC). ICC can be interpreted as the proportion of the total variance within the data that is explained by the variance between countries. The ICC is 0.135, so approximately 13.5 percent of the total variance in obesity is between countries.

I then add individual-level variables into the baseline model and estimate Model 2. Including these variables improves the model fit, according to the AIC and BIC measures. Among individual-level variables, education, age, place of residence, working and marital status display significant relationships with obesity, whereas income and gender show no significant effects. Specifically, better educated individuals are less likely to be obese. Age has a curvilinear relationship with obesity—as individuals get older, their risk of obesity increases but the rate of the increase decreases with age. Rural residents are more likely to be obese than their urban counterparts. Those who are not working have a higher chance of being obese than those who are working. There is no significant difference in obesity between single and married individuals; however, those divorced are less likely to be obese and those widowed are more likely to be obese. In contrast, income does not have a significant impact and there is no gender difference in obesity either. The effects of individual-level variables are stable across all models.

Building upon Model 2, I estimate Model 3 that further incorporates country-level variables, GDP per capita and globalization. Economic development, GDP per capita as the proxy, shows no significant effect on obesity but globalization displays a positive relationship with obesity. Globalization increases the overall risk of obesity at a global scale. Including the country-level variables in the model improves the model fit, as the AIC and BIC measures both decrease.

I further estimate Model 4 that relaxes the assumption of the same slope of income across countries and allows the slope to vary. The result from the random-slope model is consistent with that from the previous random-intercept models. In comparison with the previous models, Model 4 generates smaller AIC and BIC values, suggesting the best model fit. The slope of income is indeed different across countries, again indicating the presence of cross-country heterogeneity.

### Effect of income contingent on economic development and globalization

Across all countries there is no overall significant income-obesity relationship. Nevertheless, we cannot jump to the conclusion that income does not matter for obesity. It only indicates that income does not have a uniform impact on obesity across all countries. Next, I explore the possibility that economic development and globalization condition the effect of income on obesity, in order to test the proposed hypotheses. The interaction terms are added to the best-fit Model 4 and two more random-slope random-intercept multilevel logistic models are estimated. The results are presented in Table 3. Model 5 includes the interaction between income and GDP per capita and Model 6 contains the interaction between income and globalization. The AIC and BIC measures of Model 5 and Model 6 indicate that including the interaction terms generates better model fit than the models in Table 2.

**Table 3:** Multilevel logistic models of obesity with interactions: Testing the contingent income-obesity relationship.

	Model 5	Model 6
Fixed effects		
Intercept	−12.865*** (2.429)	−24.844*** (5.073)
Individual-level variables		
Income (in thousands)	.0023* (.0011)	.0114** (.0037)
Gender (male)	.034 (.031)	.029 (.031)
Education	−.052*** (.004)	−.053*** (.004)
Age	.142*** (.006)	.142*** (.006)
Age^2^	−.0013*** (.0001)	−.0013*** (.0001)
Residence (rural)	.080* (.033)	.081* (.033)
Working status		
Currently working	Reference	Reference
Formerly worked	.372*** (.037)	.379*** (.037)
Never worked	.310*** (.066)	.310*** (.066)
Marital status		
Single	Reference	Reference
Married	-.070 (.046)	-.072 (.046)
Divorced	-.286*** (.063)	-.286*** (.063)
Widowed	.182** (.068)	.179** (.068)
Country-level variables		
GDP per capita (in thousands)	.072 (.054)	-.010 (.013)
Globalization	.063*** (.018)	.234*** (.057)
Interactions		
Income × GDP per capita	−.000062*** (.000019)	
Income × globalization		−.00015*** (.00004)
Random effects (variance across countries)		
Intercept	Included	Included
Slope (income)	Included	Included
LR test	1048.3***	968.9***
Wald χ^2^	1105.3***	1114.5***
AIC	31064.1	31062.9
BIC	31183.4	31180.1

Notes: (1) Numbers in parentheses are standard errors; (2) from 2-tailed tests, *P < .05; **P < .01; ***P < .001; (3) under “Random Effects” the LR test reports the likelihood ratio statistic for testing the null hypothesis that the random effects (between-country variance) are zero. A significant test result indicates that it is necessary to include random effects in the modeling.

In Model 5 the coefficient of the interaction between income and GDP per capita is statistically significant and negative, so a higher level of economic development weakens the obesity-promoting effect of income. The results from Table 2, Table 3 taken together produce an interesting pattern regarding the influence of economic development on obesity. Economic development has no significant effect on the overall risk of obesity in a country, but it influences obesity through moderating the effect of income. This finding indicates that the shift toward a negative income-obesity relationship is particularly salient in affluent countries. Economic development facilitates the transition of income's obesity-promoting effect toward a more depressing one. This finding supports Hypothesis 1.

The interaction between income and globalization in Model 6 also displays a significantly negative coefficient. Hence, a country's level of globalization conditions the income-obesity relationship. The obesity-promoting effect of income becomes weaker or even turns into an obesity-depressing effect in more globalized countries. This finding lends support to Hypothesis 2. Combining the results from Table 2, Table 3, we can see that globalization not only directly elevates the risk of obesity in a country, but also indirectly affects obesity through conditioning the effect of income on obesity.

### Visualization of the Contingent Income-Obesity relationship

**Fig. 2:**
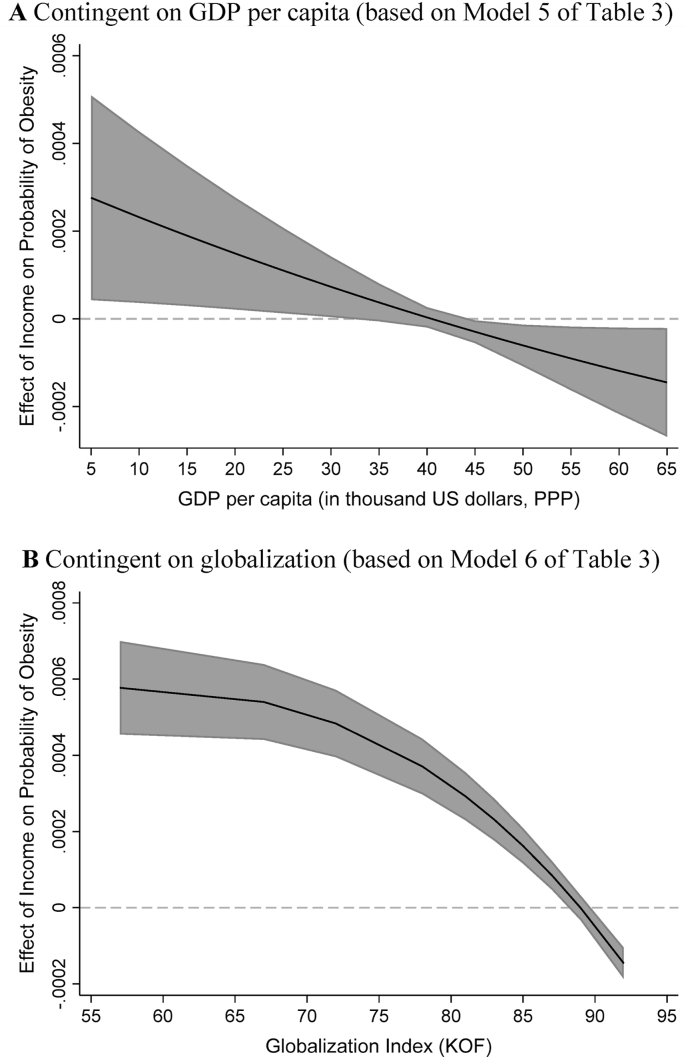
The Contingent Effect of Income (in thousand US dollars) on the Probability of Obesity (with 95% confidence intervals).

Based on the revealed interaction effects in Table 3, I create two figures in Fig. 2 that visualize the effect of income on obesity conditioned by GDP per capita and globalization, respectively, with the other explanatory variables held at their mean values. Note that when creating the figures I have transformed regression coefficients into their probability form that is more intuitive, so now the effect of income refers to the effect of income on the probability (instead of logit or log-odds) of being obese. The shaded areas in the figures represent the 95% confidence intervals.

Figure A depicts how the effect of income becomes less obesity-promoting as GDP per capita increases for an “average” country (with a mean value in the globalization and other explanatory variables). In a less developed country with GDP per capita of $5,000, the probability of being obese increases 0.028% with every $1000 increase in an individual's monthly income. In countries with GDP per capita of $10,000, $20,000, and $30,000, every $1000 increase in one's monthly income is associated with an increase of 0.023%, 0.015%, and 0.007%, respectively, in the probability of obesity. In countries with GDP per capita between $32,000 and $43,000, the probability of obesity is largely not responsive to changes in monthly income. For more developed countries with GDP per capita above $43,000, a negative association between income and the probability of obesity emerges. In countries with GDP per capita of $50,000 and $60,000, with every $1000 increase in one's monthly income, the probability of obesity decreases by 0.006% and 0.012%, respectively.

On average, income can have three differing effects on the probability of obesity in a country, conditional on the country's GDP per capita. When a country's GDP per capita is below $32,000, income shows a significantly obesity-promoting effect. With GDP per capita between $32,000 and $43,000, income displays no significant effect on obesity. Only when GDP per capita reaches $43,000 or more does income have a significantly obesity-depressing effect. Therefore, GDP per capita of $43,000 is the threshold with respect to the shift in the income-obesity relationship. In the data $43,000 is at the 85th percentile of the GDP per capita variable. At the average level of globalization, very few countries would reach the GDP per capita threshold for an obesity-depressing effect of income.

Figure B presents the changing effect of income contingent on the country's level of globalization among “average” countries. As the country gets more involved in globalization, the effect of income becomes increasingly less obesity-promoting and more obesity-depressing. For an “average” country that is average in the GDP per capita and other explanatory variables, the threshold is around the score of 90 in the KOF Globalization Index. When the country's globalization score is below 88 income has an obesity-promoting effect, and income shows no significant effect on obesity when the globalization score is between 88 and 90. With the globalization score above 90, income shows an obesity-depressing effect. In the data the score of 90 is at the 88th percentile of the globalization variable. With an average level of GDP per capita, not many countries would be able to reach the globalization threshold for an obesity-depressing effect of income.

## Conclusion and discussion

This study brings nuanced insights into the debated income-obesity relationship and highlights its contingent nature. Income does not have a uniform effect on the risk of obesity globally. Instead, both economic development and globalization are the macro-level social contexts that condition the effect of income on obesity. Greater economic development and higher levels of globalization facilitate the transition of the obesity-promoting effect of income toward a more obesity-depressing one. The cross-level interplay between macro-level social contexts and individual-level income highlights that the individual-level income-obesity relationship is embedded in broader social contexts.

Nevertheless, currently, in most countries the effect of income remains either obesity-promoting or non-significant because of the high economic development and globalization thresholds for the obesity-depressing effect of income. The findings here suggest a more modest role of economic development in shifting the direction of the income-obesity relationship in a society than that is often assumed in the literature. The reversal hypothesis popular in the existing literature asserts that the income-obesity relationship is positive in developing countries but negative in developed countries (Dinsa et al., 2012; Marteleto et al., 2017; Monteiro et al., 2004; Pampel et al., 2012). The findings here indicate that it would be difficult for economic development alone to shift the direction of the income-obesity relationship. For countries that are averagely globalized, a majority of them would show an obesity-promoting or non-significant effect of income, and very few could reach the GDP per capita threshold for an obesity-depressing effect of income. Without taking globalization into account, we may overestimate the role of economic development in reversing the income-obesity relationship.

In addition to economic development, a country's level of globalization also contributes to the shift in the income-obesity relationship in a country and should not be neglected. Globalization not only directly increases obesity in a country overall but also accelerates the country's shift toward a negative income-obesity relationship. The reversal of the income-obesity relationship observed in the existing literature is not purely due to economic development, but the role of globalization should also be noted. In the midst of ongoing globalization, a world culture that values a slim body shape has been diffusing across the globe through various forms of cultural products. Both the beauty perception and the health discourse associated with this world culture discredit and even discriminate against obesity. Wealthy individuals in highly globalized countries are particularly susceptible to this globalizing cultural influence and develop greater aversion to obesity due to their consumption of more global cultural products. Globalization thus also plays a role in reversing income's obesity-promoting effect toward an obesity-depressing one in a country.

This study has some limitations that merit further reflection. First, the ISSP data used here only cover 30 countries and may not well represent all countries in the international community. The data have a certain degree of variation among the countries covered. For instance, GDP per capita ranges from $5690 (the Philippines) to $62,077 (Norway). Overall, however, developed countries are overrepresented and less developed countries underrepresented in the data. In future data collection, a greater number of developing or less developed countries should be included. Second, some measures used in the survey are not ideal and can be improved in future surveys. In particular, despite the popularity of BMI, there are some criticisms against using BMI as a sole proxy for body fat on the basis of its inability to distinguish fat from muscle, bone, and other fat-free body mass (see Burkhauser & Cawley, 2008). In future research we should collect more data on other measurements such as waist circumference, waist-hip ratio, and body fat percentage to complement BMI. Third, it is worth noting that while economic development (GDP per capita as a proxy) is a prominent economic influence on obesity, other economic factors such as urbanization, technological change, and poverty may also play a role in shaping obesity in a society (Rosin, 2008). While they may be partly captured by GDP per capita, the effects of these other economic variables can affect obesity independently and thus warrant further investigation. Similarly, in addition to income, socioeconomic status in the relative sense, namely socioeconomic inequality, is also related to obesity (Bilger et al., 2017) and should be given closer attention in future research. For instance, the emulation or the so-called Veblen effect stemming from social comparisons may create a desire to emulate the consumption standards and behaviors (such as working and leisure activities) of those with higher socioeconomic status, which in turn has an impact on obesity in a society (Bowles & Park, 2005; Veal, 2016).

Last but not least, the findings here have implications for the social distribution of obesity. In general, income shows no effect on the risk of obesity globally, and this general non-significant effect conceals the varying effects of income across societies. Income increasingly becomes an obesity-depressing factor in more developed and highly globalized countries. The macro-level forces of economic development and globalization combine to accelerate a society's shift toward a negative income-obesity relationship. As a country becomes more developed and globalized, obesity increasingly becomes a burden of the poor and the social distribution of obesity increasingly mirrors existing social inequality. When developing obesity-related social policies, it is important to consider the contingent nature of the income-obesity relationship and tailor policies accordingly. With more economic development and globalization, the policy priority should shift toward less well-off individuals in order to prevent obesity from further enlarging social inequality.

## Sources of support

This work was supported by the Insight Grant from the Social Sciences and Humanities Research Council (10.13039/501100000155SSHRC) of Canada and the Early Career Lansdowne Scholar Award from the 10.13039/100008997University of Victoria.

## CRediT authorship contribution statement

**Min Zhou:** Conceptualization, Formal analysis, Funding acquisition, Investigation, Methodology, Writing – original draft, Writing – review & editing.

## Conflict of interest

None.
